# Workability and Mechanical Properties of Superplasticized Microfine Cement Grouts

**DOI:** 10.3390/ma15051747

**Published:** 2022-02-25

**Authors:** Fei Sha, Shijiu Gu, Yuhong Diao, Peng Liu, Deli Lou, Yan Hu

**Affiliations:** 1 College of Engineering, Ocean University of China, Qingdao 266100, China; shafei@ouc.edu.cn (F.S.); 21210913047@stu.ouc.edu.cn (S.G.); diaoyuhong1107@163.com (Y.D.); 2 School of Civil Engineering, Central South University, 22 Shaoshan Road, Changsha 410075, China; 3 Jinan Sijian Construction Group Company Limited, Jinan 250031, China; loudeli2022@163.com

**Keywords:** superplasticized microfine cement grout (SMCG), superplasticizer, rheological behavior, fresh-state property, mechanical performance, microstructure

## Abstract

Superplasticizer (SP) is essential to enhance the groutability of microfine cement (MC) in civil engineering, however, combined effects of cement type, SP type, amount of SP and water-solid ratio (W/S) on engineering performance of MC are not clear currently. In this research, workability and mechanical properties of superplasticized microfine cement grouts (SMCG) with various SPs are evaluated systematically. Three different MCs (CEM I, CEM II/B-M and CEM III/B based on EN 197-1) and four SPs (one naphthalene-based (N), one melamine-based (M) and two polycarboxylate-based (PCE)) were used to study the effect of grout formulation. The properties investigated included rheological behavior (mini-slump, flowability, time-dependent viscosity and initial viscosity), fresh-state property (bleeding, effective W/S and final setting time), mechanical performance (shrinkage, flexural strength (FS), unconfined compressive strength (UCS), and FS/UCS) and microstructure. The new method of static viscosity was adopted and viscoelasticity was evaluated. The ranges of W/S and SP content were 1.0–2.0 and 0–2.5%, respectively. The results show that the dispersion effects of SP on rheological behavior were followed by PCE, M and N in order of the influence degree. The instability, long-setting and oversaturation were easily caused by excessive SP. SP could be helpful for improving FS or bending toughness. Considering workability and mechanical performance of SMCG, the W/S is suggested to be within 1.5, the optimal amounts of N, M and PCE are recommended as 1.5–2.0%, 1.2–1.5% and 0.9–1.2%, respectively.

## 1. Introduction

The grouting technique is achieved mainly through injecting cementitious suspension or chemical liquid under pressure into cracks of concrete structures, openings of rocks, voids of soils, etc. [1,2,3]. This technique is usually adopted to restore the physical and mechanical properties of structures and/or increase impermeability and stability of strata [4,5,6]. For specific grouting engineering, the grouting material should be selected precisely based on workability, practical application requirements, long-term performance, environmental friendliness, etc. [7,8,9] Although the cost and environmental friendliness of ordinary Portland cement (OPC) are good, OPC has obvious deficiencies such as low injectability, significant filtration effect, insufficient stability, long setting time, etc. The injectability and grouting effects of OPC suspensions are poor especially for micro-cracks (≤0.5 mm) and small voids [10,11,12]. Although the injectability of chemical grout is high, they were not environmental friendly especially after the acrylamide poisoning incident in 1974 in Fukuoka. They present poor bonding in moist environment and insufficient long-term performance (chemical stability and creeping) [13,14]. Moreover, the practical operations of chemical grouts are complex, the reaction rate is not easy to control, and they are extremely expensive. Therefore, the large-scale application of chemical grout has great limitations especially under the current big backgrounds of green sustainable development, water conservancy and ecological environment restoration.

To inject the microcracks of concrete or rock structures and small voids of fine sands effectively, microfine cements (MC) with related techniques are widely applied in replacing chemical grouts [9,11,14,15]. MC is defined by ACI Committee 552 (Geotechnical Cement Grouting) by diameters of all particles smaller than 15 μm, MC is described as *d*_95_ < 16 μm by the International Society for Rock Mechanics, and MC is characterized as *d*_95_ < 20 μm and Blaine fineness ≥ 800 m^2^/kg based on EN 12715 [16]. As the fineness increases, the adsorption amount of water by solid phase particles increases, thereby reducing the fluidity of slurry under the same water-solid ratio (W/S) [17]. Therefore, to acquire workable grout with high flowability, spreading ability and penetration ability, it is essential and important to apply superplasticizer (SP) because of the high specific surface area of MC [14,18,19,20].

The naphthalene-based SP (hereinafter called N) and melamine-based SP (hereinafter called M) are the typical second generation of SP, and they are commonly manufactured from sulfonated naphthalene or melamine formaldehyde. Polycarboxylate-based SP (hereinafter called PCE) is called the third generation SP. The water reducing mechanism of N and M was mainly reflected on the effects of adsorption and electrostatic repulsion [21,22]. Meanwhile, the combined effects of electrostatic repulsion and steric hindrance of PCE ensured its good water dispersion stability [23]. Many studies presented that the carboxylic groups act as anchors to adsorb on the surfaces of cement particles, and the flocculation and hydration of charged colloidal grains were weakened by the steric hindrance of side chains [24,25,26].

In this research, workability and mechanical properties of superplasticized microfine cement grouts (SMCG) with N, M and PCE were evaluated systematically. The purposes of this experimental study were as follows: (1) To study the combined effects of different types of SP (N, M, PCE), SP content, cement composition and key W/S on engineering properties of SMCGs, which is rare for current studies; (2) To evaluate rheological behavior (mini-slump, flowability, viscosity) and fresh-state property (bleeding, effective W/S and final setting time); (3) To determine main mechanical strength (shrinkage, flexural strength (FS), unconfined compressive strength (UCS) and FS/UCS) and microstructures of SMCGs with various SP contents, therefore, providing missing key data in existed references; (4) To analyze comparatively and evaluate the compatibility of SPs and MCs, thus, giving suggestions for formulation optimization of SMCG in applications.

## 2. Significance

The effects of SP on rheological performance of ordinary cementitious composites at lower W/S (<1.0) were presented frequently. However, with the increasing of water reducing efficiency of SP, the generational upgradation of SP, the increasing of fineness and W/S (≥1.0), scientific findings about the synergistic effects of cement composition, SP type, content of high-efficiency SP and W/S on performance of SMCG, compatibility of SP and MC, and systematic optimization of SMCG with SP are lacking. Meanwhile, it is urgent and essential to ensure the quality of SMCG suspensions and provide key property data that are reproducible on the field. This experimental study was designed to fill this gap. This research presents a comparative investigation of various SMCGs containing different types of new SP. These grouts were formulated in various projects, such as reinforcements of concrete structures, anti-seepage or strengthening of fractured rock masses and sandy strata, consolidation of bridge piles or soil foundations, restoration of maritime structures, etc. The compatibility of MC and new generation SP and systematic optimizations are new and vital. The comprehensive performance evaluation (rheological properties, fresh states, mechanical properties and microstructures) of such SMCGs with various SP has not been presented previously.

## 3. Materials and Methods

### 3.1. Raw Materials

Portland MC (CEM I), Portland-based MC (CEM II/B-M) and blast-furnace slag-based MC (CEM III/B) from Sunnsy Company were selected as three typical types of cements in Shandong Province in China, considering multiple factors such as performance, practicability and cost. They all belong to ASTM I cement. Their chemical compositions and raw materials are shown in Table 1. The distinctions in cement type are reflected on the expected differences in raw material and oxide composition. The amounts of clinker and calcium of CEM I cement are clearly higher compared with those of CEM II/B-M and CEM III/B. The amount of blast-furnace slag (BFS) in CEM III/B is much higher, and it has the highest concentration in silica and alumina and the lowest concentration in calcium.

**Figure 1:**
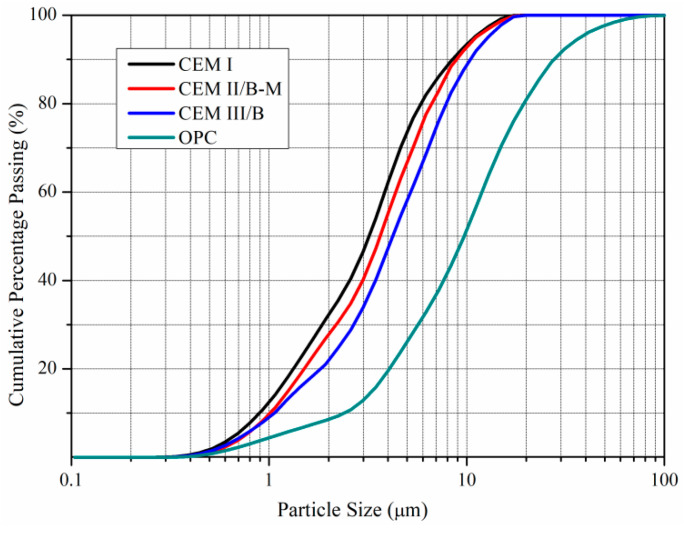
Particle size distributions (PSD) of MCs and OPC.

These MCs were pulverized by superfine dry grinding in Shandong Province in China. Their particle size distributions (PSD) characteristics are shown in Figure 1. The grain-size characteristic parameter and Blaine specific surface values of MCs and OPC are presented in Table 2. The *d*_50_, *d*_95_ and *d*_100_ of three MCs ranged among 3.21–4.22, 10.92–12.81 and 20.02–20.50 μm, respectively. According to the PSD parameters, the injectability of MC could be better than that of OPC. The micro cracks (<0.5 mm) of concrete or rock structures should be injected with MCs to guarantee effective filling and complete cementation inside the micro cracks [27]. The specific surface area of MC falls into the following order: CEM I > CEM II/B-M > CEM III/B.

### 3.2. Superplasticizer

**Figure 2:**
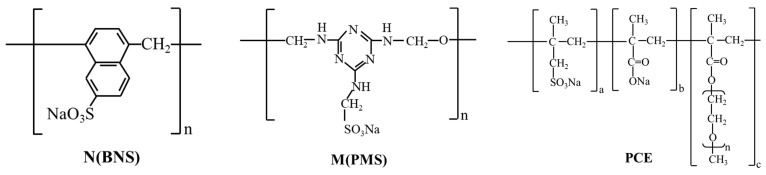
Chemical molecular structure of superplasticizers (N-BNS, M-PMS and PCE).

Three types of commercial SPs (N (Wanshan), M (Sika), PCE (Baochen)) from Jinan of Shandong Province in China were selected to prepare SMCGs. Their chemical molecular structures of SP are shown in Figure 2. The main properties and dispersing classification of different SP are presented in Table 3.

### 3.3. Preparation of SMCG Suspensions

The temperature of raw materials was 25 ± 2 °C. As for fresh slurries with various SP, the SP (relative to the mass of MC) was firstly mixed with the designed water by a high turbulence mixer (Yongkangmeinuo) from Jinan of Shandong Province in China at 1000 rpm for 2 min. Then the required MCs were added and the fresh slurries were stirred continuously at 1000 rpm for 3 min. For the fresh suspensions without SP, the agitation speed was still 1000 rpm, however, the agitation time was 5 min to guarantee dispersibility. The W/S ranges were chosen as 1.0–2.5 (1.0, 1.2, 1.5, 2.0 and 2.5). The contents of N, M and PCE (relative to the mass of cement) were selected as 0.5–2.5% (interval variation value of 0.5%), 0.3–2.1% (interval variation value of 0.3%), and 0.3–1.5% (interval variation value of 0.3%), respectively. The saturation point or transitional zone is expected to be observed with the increased amount of SP.

### 3.4. Experimental Approach

**Figure 3:**
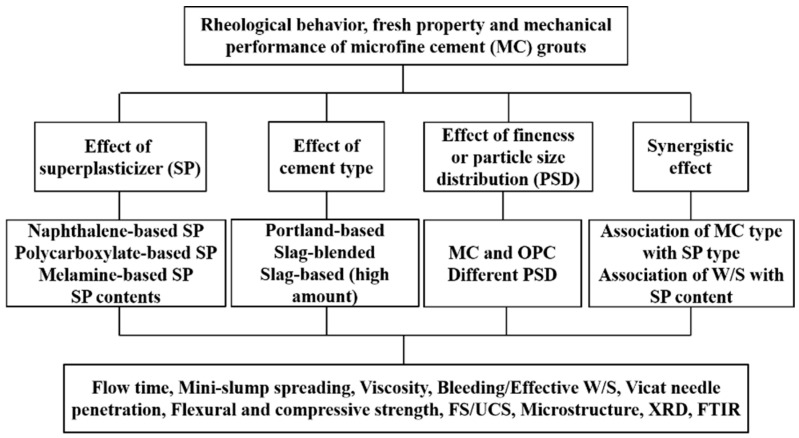
Experimental formulation and approach.

Figure 3 shows the experimental formulation and approach of this study. The effects of cement type, upgraded SP type and grout formulation (association of W/S, SP type, SP content and PSD) on the performance of SMCG were evaluated. In order to evaluate flowability and spreading ability of fresh SMCG slurries, the Marsh cone flow time and mini-slump spreading diameter were combined and performed systematically. These measurements are effective methods to estimate fluidity performance of fresh slurries prepared at site works. The mini-slump measurement can be calculated as the average spreading diameter in a plate, the cone of mini-slump is similar to that cone of concrete (ASTM C-143) [28]. Specifically, the sizes of height, bottom diameter and top diameter were 60, 36 and 60 mm, respectively. The elapsed time of specific volume of fresh suspension through the Marsh cone can be defined as the Marsh cone flow time (ASTM C 939) [29]. The internal orifice diameter of the Marsh cone is 4.8 mm, and the 1500 mL fresh slurry was filled in the Marsh cone. The elapsed time was recorded when the 946 mL of fresh suspension had just completed the flow in this study. In comparison, the flow time of 946 mL water was about 26 ± 0.5 s.

The vibration viscometer of SV-10 (A&D Company controlled corporation in Tianjin in China) was adopted to measure the time-dependent viscosity and initial viscosity, the viscosity investigated in this study was the static viscosity, which is a combined effect of elastic and viscous performance [20,30,31]. Unlike rotational or other vibration viscometers [32,33], the general measurement scope is sustaining, sensitive and wide (0.3–10000 mPa·s), this type viscometer is not related to shear rates of fresh suspensions and it can also work if the shear rate is not known or cannot be associated at site works. This viscometer is suitable for measuring the time-dependent viscosity or viscoelastic behavior of non-Newtonian fluids such as suspensions [34,35]. As for the test principle of SV-10, the oscillator is actuated by electromagnetic power and their vibration frequencies are the same. A sympathetic vibration composed of tuning fork and oscillator plays the role of sensitive sensor. The electric current functions as exciting force, and the key coefficient is acquired through determining the quantitative relationship between the electric current and viscidity.

Bleeding capacity is calculated as the ratio of ΔV/V_0_, the V_0_ is the initial volume of fresh suspensions and the ΔV is the volume of bleeding water. The graduated cylinder (250 mL) was filled with fresh SMCG suspensions, and the volume of bleeding water was recorded after 2 h of sedimentation. Based on bleeding rates, the effective W/S was calculated as follows:

Effective W/S = (1 − *C_B_*) × Initial W/S (1)

where *C_B_* is the coefficient of bleeding volume. The fresh suspension is regarded as ‘‘stable’’ if its bleed capacity was not over 5% after 120 min [16]. The final setting time was adopted to evaluate the engineering setting performance, because the secondary or later sequential drillings must await the final setting of grouts in the initial grouting holes and reinforced structures. The Vicat needle apparatus (Luda) from Shanghai in Shandong Province in China was used to determine the final setting time. The final setting time was determined when the penetration height of the needle was less than 0.5 mm. Based on ASTM Standard C531 [36], the shrinkage performance of SMCG with different SP and cement type was evaluated at the W/S of 1.0. The dimension of SMCG mortar specimens was 250 × 25 × 25 mm. The curing conditions were 30% R.H, and the curing ages were 3, 7, 14, 28, 56, 91 and 180 days.

There is little research about flexural strength (FS) and unconfined compressive strength (UCS) of SMCGs, especially SMCGs with different cement types, SP types and SP contents. The FSs were determined to give practical references for such SMCGs. The size of cubic specimen mold for FS and UCS was 40 × 40 × 160 mm. If there was obvious bleeding, the bleeding water was removed until there were merely hardened grout stone bodies. On the basis of GB/T 17671-1999 [37], the 3-day, 7-day and 28-day FS and UCS tests of hardened grouts were conducted, and the loading rate was 2 mm/min. To evaluate the brittleness of SMCG grout stone bodies, the FS-UCS ratio (FS/UCS) of MCG was calculated and analyzed comparatively. Small broken pieces of hardened SMCGs were kept in ethyl alcohol. The D8 ADVANCE type X-ray diffraction analyzer (Bruker, in Karlsruhe in Germany) was used to measure the hydration minerals, the scanning angle and scanning speed were 5–60° and 4–5°/min, respectively. Fourier transform infrared spectroscopy (FTIR) was performed through a spectrometer (Thermo Fisher in Waltham, MA, USA) on SMCG samples. The spectral analysis was performed in the range of 400 to 4000 cm^−1^, with a spectral resolution of 1 cm^−1^. The microstructure of MCGs was studied by using a scanning electron microscope (SEM, (ZEISS in Oberkochen in Germany)).

## 4. Results and Discussions

### 4.1. Spreading Ability and Flowability

The spreading or propagation ability into cracks or soil pores of fresh grouts can be characterized macroscopically by the variations of mini-slump. The Marsh cone flow time can represent the flowability during mixing or pumping in field works. The flow time is proportional to the friction between slurries and cone sides. 

Table 4 presents the mini-slumps and flow times of fresh MCs (without SP) under different W/S. With similar PSD values, the effects of W/S on mini-slump were more significant than cement type, and the effects of cement type on flow time were not obvious, especially when W/S was over 2.0. Compared with OPC, the MC adsorbed more water due to their larger specific surface areas, therefore, their flowability and spreading ability were low. The mini-slumps of MCGs at W/S of 2.0 (295–299 mm) were still much less than those of OPC at W/S of 1.0 (328 mm). The flow times of MCs at W/S of 2.0 (28.22–29.19 s) were lower than those of OPC at W/S of 1.0 (30.19 s). This variation difference might be because: the slurries are subjected to a high shear rate in Marsh cone flow tests, while low shear rates are generated during mini-slump tests, and the slurries are likely more susceptible to the friction or resistance. The spreading ability and flowability became better when the slag content became larger, in comparison, Portland-based cement (CEM I) with higher content of clinker tended to decrease the fluidity of slurry.

**Figure 4:**
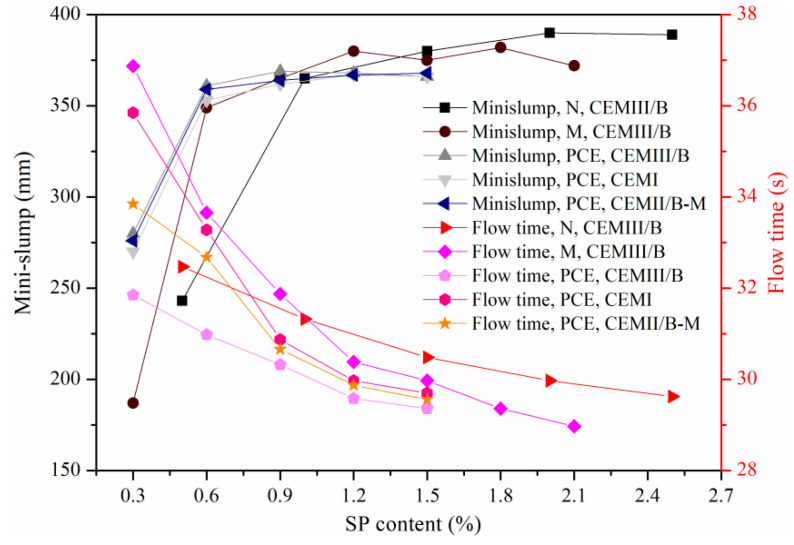
Effect of SP and cement type on mini-slumps and flow times of SMCG at the W/S of 1.0.

Figure 4 shows effects of SP and cement type on mini-slumps and flow times of fresh SMCG, and the W/S of 1.0 is selected because it is usually applied in the field works. The mini-slump increased significantly when the amounts of N, M and PCE reached or exceeded 0.5%, 0.6% and 0.6%, respectively. For example, the growth degrees of mini-slump for CEMIII/B were 50.21%, 86.63% and 28.93% when the increase ranges of N, M and PCE were 0.5–1.0%, 0.3–0.6% and 0.3–0.6%, respectively. Although the growth degrees of suspensions with PCE were relatively low, their mini-slump values were generally high with low PCE contents. For instance, the mini-slumps of CEMIII/B suspensions were 243, 187 and 280 mm when the amounts of N, M and PCE were 0.5%, 0.3% and 0.3%, respectively. At W/S of 1.0, the effects of SP type and SP content on mini-slumps were more significant than those of cement type. The increase in degree of mini-slump was little when the amounts of N, M and PCE exceeded 2.0%, 1.2% and 0.9%, respectively. It illustrated that the saturation point might exist, the increase degrees of mini-slumps became little and it was unnecessary to add the highest amount of SP to obtain satisfied mini-slumps of SMCG. The little or negative increase in mini-slumps at higher SP contents might be due to the relatively obvious frictions at low shear rates. The flow times of SMCG with less PCE can be close to those of SMCG with more N and M, and the decrease effects of PCE on flow time were more obvious. For example, the flow time of CEMIII/B with 0.9% PCE was 30.32 s, while those with 1.5% N and 1.2% M were 30.48 s and 30.38 s. When the W/S was mixed at 1.0, the effects of cement type on flow time were obvious when PCE contents were relatively low (≤0.6%), and the SP content was the dominant factor. At higher SP contents, the decrease degrees of flow time were relatively obvious compared with the increase degrees of mini-slumps, the flow time decreased all the while with the increase in SP content, and the saturation point was not observed easily. It might be because the flow time can reflect the flowability and viscosity well at high shear rates, meanwhile, the influence of friction was less significant. With the increase in slag contents in SMCG, it was observed that the flow time decreased and mini-slump increased. At the W/S of 1.0, the mini-slumps and flow times of SMCG were relatively satisfactory when the amounts of N, M and PCE were 1.5–2.0%, 1.2–1.8% and 0.9–1.5%, respectively. 

### 4.2. Viscosity Variation and Initial Viscosity

In this study, the viscosity is the time-dependent ‘static viscosity’. In the mini-slump and Marsh cone flow tests, the shear rates applied to suspensions were not known. The time-dependent viscosity can provide related references if shear rates are not clear and the effects of time need to be determined at site works. Initially, fresh suspensions were prepared with three cement types at initial W/S of 1.0, 1.5 and 2.0.

**Figure 5:**
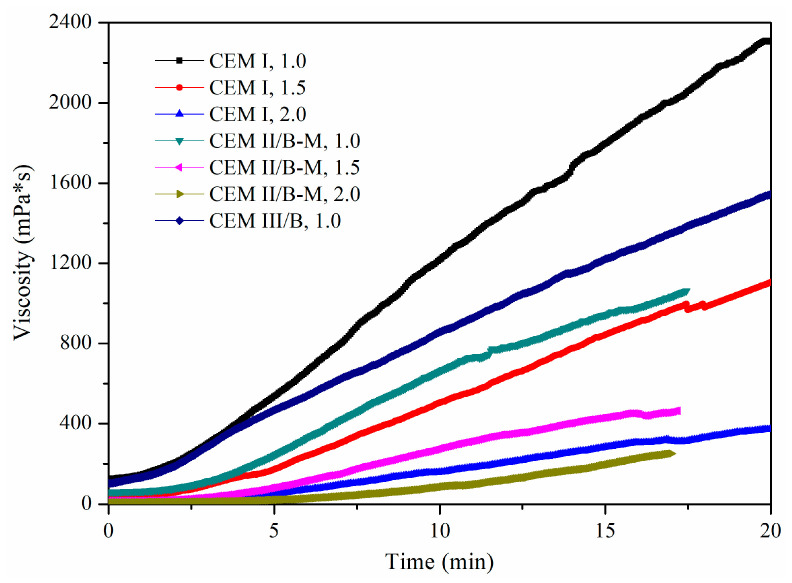
Effect of cement type and W/S on viscosity variation of fresh SMCG (0% SP) within initial 20 min.

Figure 5 shows combined effects of cement type and W/S on viscosity of fresh SMCG without SP. Within initial 20 min, the viscosity ranges of CEM I were about 126.02–2363.17, 24.28–1139.96 and 8.92–391.33 mPa·s at W/S of 1.0, 1.5 and 2.0, respectively. The related viscosity ranges of CEMII/B-M were about 60.37–1062.02, 15.85–463.72 and 8.50–251.04 mPa·s at W/S of 1.0, 1.5 and 2.0. The time-dependent viscosity of fresh MCG was low and relatively satisfactory at the W/S of 2.0, which was consistent with the results of mini-slumps and flow times in Table 4. Without SP, the viscous behavior of SMCG was prohibitively high if the W/S was less than 2.0, and the viscosity of microfine slurry might meet the requirements of groutability when the W/S exceeded 2.0. It might be because the high adsorption amount of water by solid particle of SMCG increased observably with the increase in fineness. The viscosity of CEM I MC was higher than that of CEM II/B-M MC in general, which was in accordance with the performance of mini-slumps and flow times. It might be because a higher content of clinker hydrates more violently and tends to increase viscosity in early hydration periods.

**Figure 6:**
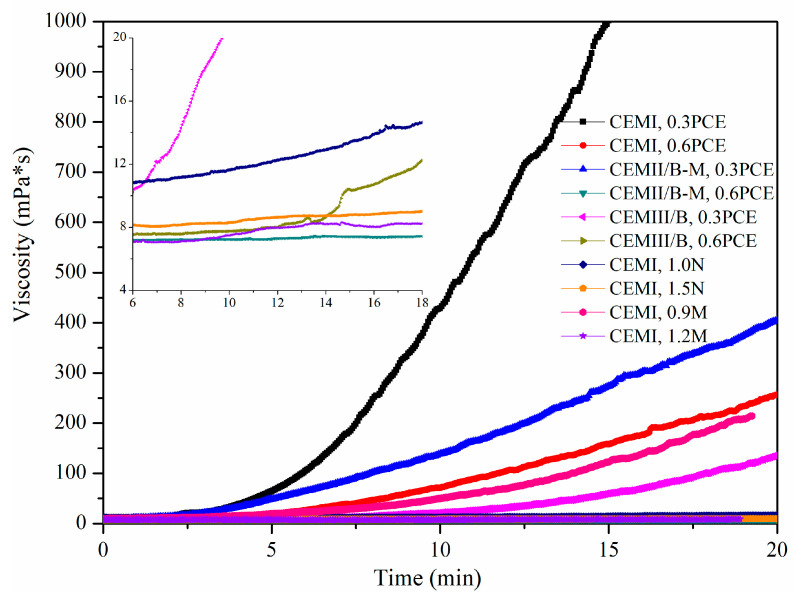
Effect of SP and cement type on viscosity variation of fresh SMCG at the W/S of 1.0.

Figure 6 shows effects of SP and cement type on viscosity variation of fresh SMCG at the W/S of 1.0. The effects of SP type and SP content on the viscosity of fresh SMCG were more obvious than those of cement type. Within 15 min, the viscosities of CEM I, CEMII/B-M and CEMIII/B grouts with 0.3% PCE were about 7.74–994.68, 7.96–405.07 and 7.88–135.59 mPa·s, respectively. While those with 0.6% PCE were 7.18–256.43, 7.17–7.55 and 6.87–13.12 mPa·s within 20 min. The decrease degrees of viscosity from 0.3% PCE to 0.6% PCE were much higher than those affected by different microfine cement type. The Portland-based cements with more clinker tends to have higher viscosity under the same condition. To improve flowability, pumpability or injectability of MC, the low viscosity and excellent viscous behavior are expected. As for CEM I and W/S of 1.0, the amounts of N, M and PCE should be no less than 1.5%, 1.2% and 0.6% to ensure low viscosity. The viscosity decreasing effect of PCE was the most obvious and that of N was the least. The adsorption of SP destroys the flocculated structures of cement-based slurry, then the water is released from the flocculated structures, and the viscous behavior and spreading ability of SMCG have been improved accordingly. The amount of SP should not be over to avoid prohibitive bleeding, supersaturation, slow condensation, serious instability, etc. The low initial viscosity can ensure satisfied initial spreading ability and flowability of fresh slurries. Therefore, it is necessary to determine and assess the related initial viscosity.

**Figure 7:**
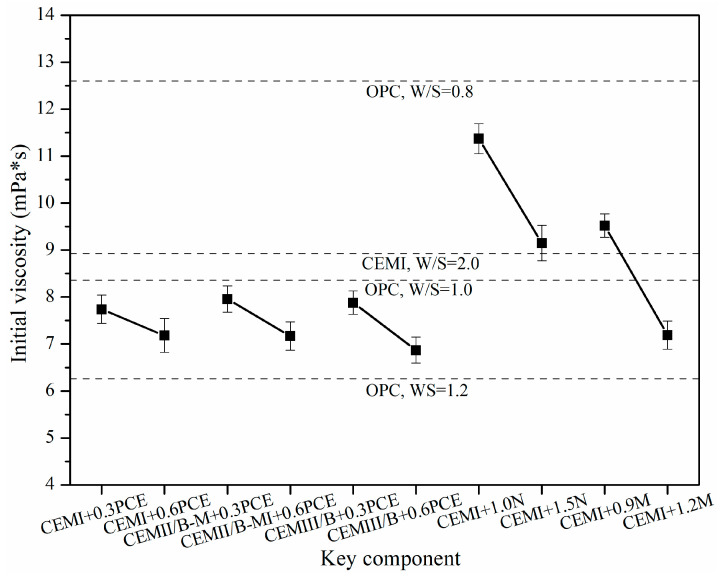
Effect of SP and cement type on the initial viscosity of fresh SMCG at the W/S of 1.0.

Figure 7 shows effects of SP and cement type on the initial viscosity of fresh SMCG at the W/S of 1.0. There are few references about the combined effects of SP and MC type on this initial viscosity, and these related values are compared with each other. When the amount of PCE increased from 0.3% to 0.6%, the decrease degrees of initial viscosity were about 0.72‰ (7.74−7.18 mPa·s), 0.44‰ (7.50−7.17 mPa·s) and 12.82‰ (7.88−6.87 mPa·s) for CEM I, CEMII/B-M and CEMIII/B SMCG, respectively. Although there existed some differences of decrease degree among different cement types, the differences among initial viscosity were not large, and the initial viscosities of CEM I were generally high. When the amounts of N and M increased from 1.0% to 1.5% and 0.9% to 1.2% for CEM I, the decrease degrees of initial viscosity were about 19.53‰ (11.37−9.15 mPa·s) and 2.45‰ (9.52−7.19 mPa·s), respectively. The initial viscosities of three MCGs with minimum PCE content (0.3–0.6%) at W/S of 1.0 (7.88−6.87 mPa·s) were lower than those of OPC at W/S of 1.0 (8.36 mPa·s) and close to those of OPC at W/S of 1.2 (6.26 mPa·s). The initial viscosities of CEM I MCG with 1.0–1.5% N at W/S of 1.0 were higher than those of CEM I MC at W/S of 2.0 (8.92 mPa·s) and OPC at W/S of 1.0 (8.36 mPa·s). The initial viscosity of CEM I with 1.2% M was lower than those of OPC at W/S of 1.0 and close to those of OPC at W/S of 1.2, however, that with 0.9% M was higher than those of CEM I MC at W/S of 2.0 and OPC at W/S of 1.0. Compared with the influence of component on initial viscosity, the W/B is the key controlling element. To obtain relatively satisfied initial viscosity of SMCG at the W/S of 1.0, the amounts of PCE, N and M are suggested to be no less than 0.6%, 1.5% and 1.2%, respectively. Based on the time-dependent viscosity variation and initial viscosity values, the water reduction or dispersion effects of SP were followed by PCE, M and N. 

The N and M series are considered to be rigid rod-shaped molecules, their adsorption morphologies are accumbent straight-chains [38]. The comb-shaped side chains were the main adsorption forms of PCEs [39]. The function mechanism is mainly reflected on the electrostatic repulsion effect. The DLVO theory holds that the charged colloidal particles are the results of the interactions among electrostatic repulsion when the electric double layer overlaps and the van der Waals force forms among cement particles. The adsorption of SP changes the charge distribution on the surface of the cement particles, reduces the thickness of the electric double layer, and increases the dynamic potential, thereby improving the dispersion between the particles. As for MCG with PCE, many studies found that the DLVO theory could not support the related function mechanism of PCE, and the steric hindrance effect was also accepted [40,41]. The main chain of the PCE molecule is firmly adsorbed on the surface of the MC particles, the side chains with lower density are surrounded by cement particles to form contacted adsorption layers. Due to the flexibility of side chains, the thickness of adsorbed layer might decrease, and the distance among mineral grains decreased. Accordingly, the effects of electrostatic repulsion among MC particles might be enhanced. The combined effects of electrostatic repulsion and steric hindrance effectively enhance the mutual repulsion among easily absorbed MC grains. The synergetic function mechanism of PCE is different from the mechanism of N and M, thus having better dispersing ability and water reducing effect [42]. This explanation was in in accordance with the good spreading ability, flowability and viscosity of MCG with low PCE contents in this study.

### 4.3. Bleeding Capacity and Effective W/S

Table 5 presents bleeding rates of SMCG slurries with various SP, cement type and W/S. The amounts of PCE, N and M were selected as 1.2%, 2.0% and 1.5%, and the W/Ss were chosen as 1.0, 1.2, 1.5 and 2.0. In this study, the “stable” slurry was determined when the bleeding capacity was not over 5.0% after 2 h from preparation based on EN 12715. The stable slurries ought to be preferred in good grouting practices, because there existed an appreciable quantity of unfilled micro-cracks or soil voids due to bleeding. The less the bleeding capacity, the higher the stone ratio of fresh SMCG slurry.

In Table 5, bleeding capacity increased with the increase in W/S. The SMCG slurries were stable at the W/S of 1.0 and 1.2, regardless of the addition amounts of different SP in this study. Without SP, the slurries were stable at the W/S of 1.5, and they generally became unstable when W/S exceeded 1.5. This result is generally in accordance with the finding that MCG is stable for W/S until 1.6. When the W/S reached or exceeded 1.5, the addition of SP (1.2% PCE, 2.0% N and 1.5% M) generally resulted in the increase in bleeding capacity. The bleeding difference between OPC and MC was significant, the bleeding seems to be influenced more observably by PSD or fineness than the cement type. The bleeding rates of CEM I grouts with 2.0% N and 1.5% M were higher than those with 1.2% PCE; this might be because the amounts of N and M were relatively high.

**Figure 8:**
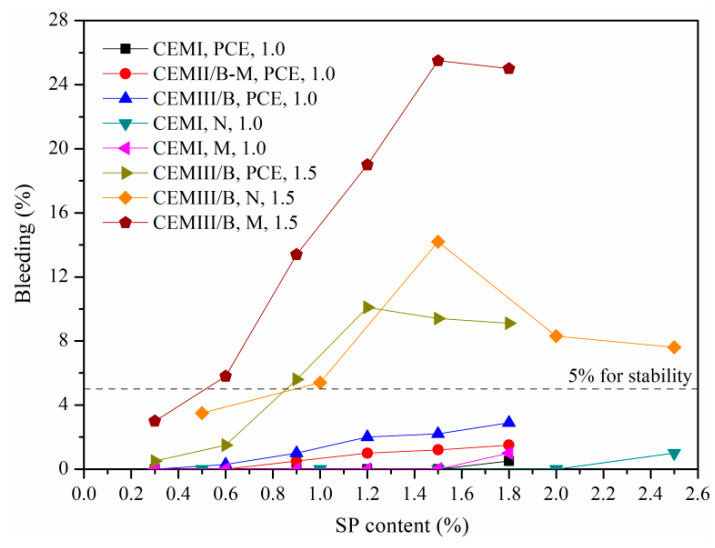
Effect of SP content and cement type on bleeding rates of fresh SMCG at W/S of 1.0 and 1.5.

Figure 8 presents the effect of SP content and cement type on bleeding rates of fresh SMCG slurries at W/Ss of 1.0 and 1.5. At the W/S of 1.0, the bleeding rates were all less than 5%, regardless of SP type and SP content. At the W/S of 1.5, the CEM III/B was selected to illustrate the effect of SP content on bleeding. When the amounts of N, M and PCE exceeded 1.0%, 0.6% and 0.9%, the bleeding rates increased observably and the fresh CEM III/B MCG started to become unstable rapidly. To ensure the stability of slurries, the SP content should not be very high when the W/S exceeded 1.5. When the W/S is exceeded, the amount of SP should be not too high to ensure the good stability and avoid oversaturation phenomena of SMCG slurry. At the initial W/S of 1.5, the M tended to be unstable more easily when the SP content increased. At the initial W/S of 1.0, the bleeding phenomenon was not obvious, and the amounts of N, M and PCE could attain 1.8–2.0%.

**Figure 9:**
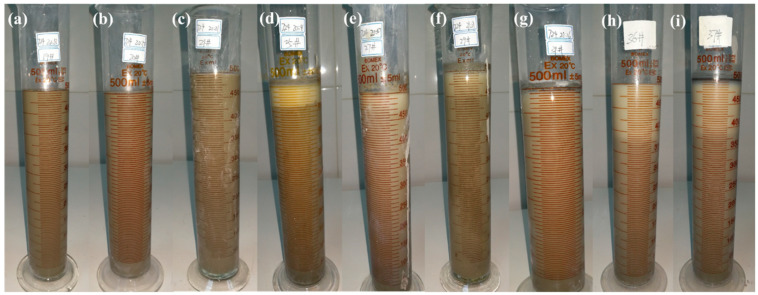
Typical bleeding photos of the fresh slurries (**a**) CEMIII/B, 1.0, 0.6%M (**b**) CEMIII/B, 1.0, 1.2%M (**c**) CEMIII/B, 1.0, 1.8%M (**d**) CEMIII/B, 1.5, 2.0%N (**e**) CEMIII/B, 1.5, 2.5%N (**f**) CEMIII/B, 1.5, 1.2%PCE (**g**) CEMIII/B, 1.5, 1.5%PCE (**h**) CEMIII/B, 1.5, 1.5%M (**i**) CEMIII/B, 1.5, 1.8%M.

Figure 9 shows typical bleeding photos of the fresh slurries at W/Ss of 1.0 and 1.5. There was almost no bleeding water for CEMIII/B SMCG slurries with W/S of 1.0, regardless of the amount of M. While at the W/S of 1.5 and 1.5–1.8% M, the oversaturation phenomenon or segregation layer was obvious. The 1.5% content of M might have been the transitional point of saturation, especially after high-speed mixings. At the W/S of 1.5, the saturation phenomenon and segregation layer were observed for 1.5–2.0% N or 1.2–1.5% PCE. When the W/S exceeds 1.5, the amounts of N, M and PCE are suggested to be within 2.0%, 1.5% and 1.5% to avoid oversaturation.

**Figure 10:**
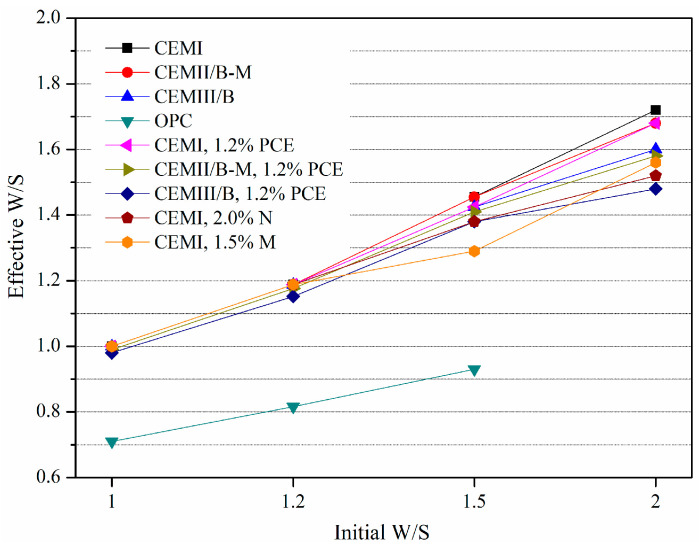
Effect of SP, cement type and initial W/S on the effective W/S of fresh SMCG slurry.

The effective W/S is necessary and helpful for understanding hydration efficiency and mechanical property difference caused by bleeding phenomenon. Figure 10 shows the effect of SP, cement type and initial W/S on the effective W/S of fresh SMCG slurry. The effective W/S is closely related with bleeding capacity under various initial W/S. For stable slurries at initial W/S of 1.0 and 1.2, the effective W/S was almost identical to the initial W/S. With the increase in initial W/S, the difference between effective W/S and initial W/S increased accordingly. The fineness is still the key controlling factor compared with the effective W/S of SMCG and OPC, and the differences of effective W/S among different MCs (without SP) were little. The SP type, SP content and W/S should be combined to shorten the difference between effective W/S and initial W/S, especially under the conditions of high SP content and high initial W/S.

### 4.4. Final Setting Time

**Figure 11:**
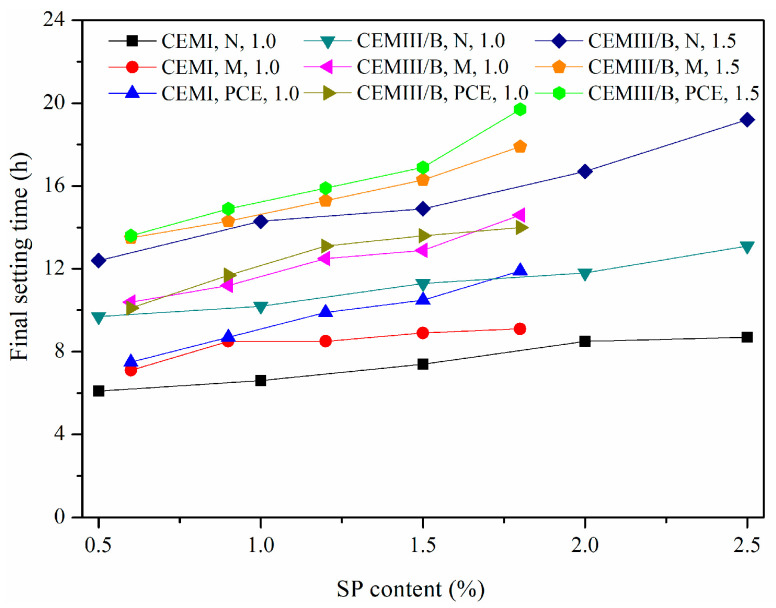
Final setting times of fresh SMCG with different SP, cement type and W/S.

Determination of setting time is practical for grouting projects, because the subsequent drilling must wait for the final setting of grouts in the previous grouting holes. Figure 11 shows the final setting times of fresh SMCG with different SP, cement type and W/S. The appropriate final setting time is advised because rapid setting can lead to opening cracks or noneffective filling during the blocking process and long setting may be disadvantageous due to probable washout, useless running slurry or freezing below 0 °C. The final setting time increased generally with the increase in SP content, regardless of SP type. Although the effect of W/S on the final setting time was obvious, the effect of SP content should not be neglected. The final setting times of SMCG with PCE tend to be larger than those with N and M, this might be because the delaying of hydration reaction is more obvious and their plasticity is enhanced markedly. Meanwhile, the lower bleeding rates and more water retaining capacity of MC with PCE might enhance the final setting time. The final setting times of SMCG are larger than 4 h, and this can ensure that the final setting time is not rapid. With the increase in slag content or decrease in clinker content, the final setting time increased with different degrees. At the W/S of 1.5, the final setting times of CEM III/B SMCG with 0.5–2.0% N, 0.6–1.8% M and 0.6–1.8% PCE were about 12.4–19.2 h, 13.5–17.9 h and 13.6–19.7 h, respectively. These final setting times were less than 24 h, it might be due to the relatively high hydration rates generated by fine cement grains and high specific surface areas.

### 4.5. Stability of Stone Body

**Figure 12:**
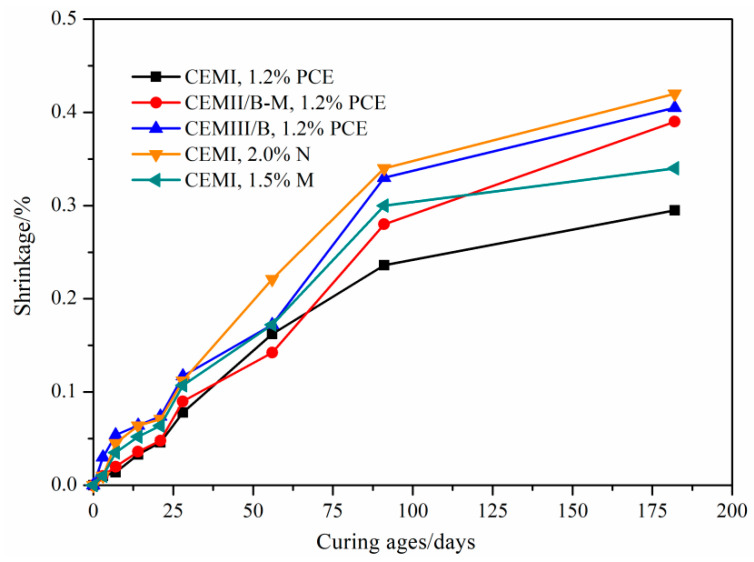
Shrinkage of SMCG stone bodies with different cement type, SP type and SP content at 30% R.H.

Due to bleeding, the SMCG can set at various speeds throughout their bodies, and the shrinkage is easily caused especially for the fine cement grains. In grouting practice, the lack of moisture or low ambient humidity can result in reopening of cracks and decreasing interface bonding strength. The shrinkage of SMCG stone body is presented in Figure 12 to evaluate the dimensional stability stone body. In Figure 12, all SMCG with different SP and cement type shrank at 30% R.H. With 1.2% PCE, the shrinkage values of slag-based grout (CEM III/B) were highest, followed by slag-blended grout (CEM II/B-M) and Portland-based grout (CEM I). The range of shrinkage range of SMCG was 0.30–0.41% within 180 days. The shrinkage rates of CEM I SMCG with 2.0% N and 1.5% M were higher than those with 1.2% PCE, and this might be due to the lower bleeding capacity and higher capillary water contents of SMCG with 1.2% PCE. The hardened SMCG requires moisture in a period of time to fulfill the main hydration process, and the time is not merely some hours after mixing or preparation. The loss of capillary water of fine cement grains might be the main reason for the shrinkage in dry environments [43]. In dry environments, some studies verified that water molecules controlled by capillary tension in small capillaries (5–50 nm) are easily moved away through the function of evaporation, and this might lead to the shrinkage of hardened stone body [44,45]. In conclusion, the shrinkage is closely related with the environmental humidity, cement type, SP type, SP content and setting rates.

### 4.6. Flexural and Unconfined Compressive Strength

**Figure 13:**
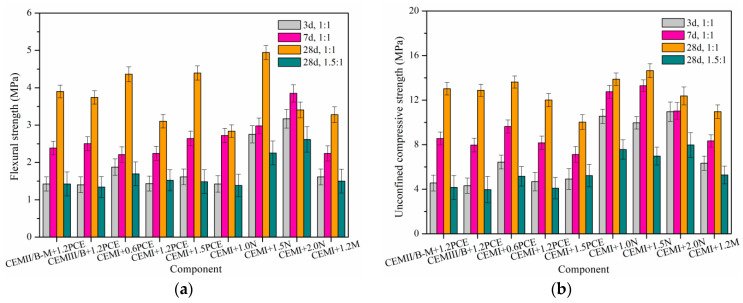
Flexural and unconfined compressive strengths of SMCG with key formulation (cement type, SP type, SP content and W/S). (**a**) Flexural strength; (**b**) Unconfined compressive strength.

The studies about combined effects of MC type, SP type, SP content and W/S on the flexural strength (FS) and unconfined compressive strength (UCS) of SMCG are rare at present. Figure 13 shows the FS and UCS of SMCG with key components at W/Ss of 1.0 and 1.5. The FS of SMCG stone body was related closely with the SP type, SP content, curing day and W/S. There were differences among variation characteristics of 3-day, 7-day and 28-day FSs. The 28-day SMCG stone bodies have matured and the 28-day FSs were analyzed emphatically. In comparison, the 28-day FSs of CEM I SMCG were about 2.91 and 1.33 MPa when the W/Ss were 1.0 and 1.5, respectively. With 1.2% PCE, the 28-day FS was followed by CEM II/B-M, CEM III/B and CEM I, the 28-day FS range of SMCG was approximately 3.1–3.9 MPa, which were generally higher than that of CEM I SMCG without SP. To obtain high 28-day FS, there existed relatively optimal amounts of N and PCE. For example, the 28-day FSs of CEM I SMCG with 1.5% N and 1.5% PCE were 4.94 and 4.39 MPa, respectively. The 28-day FS of CEM I SMCG decreased when the N content increased from 1.5% to 2.0%, and the high amount (≥2.0%) of N might be adverse for the 28-day FS of CEM I stone bodies. Although the 28-day FS (3.28 MPa) of CEM I SMCG with 1.2% M was lower than that with 1.5% N or 1.5% PCE, it was higher than that of CEM I SMCG without SP. It was also observed that the 28-day FS of various SMCG at the W/S of 1.5 was generally lower or close to the 3-day FS of SMCG at the W/S of 1.0. For example, at the W/S of 1.5, the 28-day FSs of SMCG with 0.6–1.5% PCE, 1.0–2.0% N and 1.2% M were about 1.48–1.69, 1.38–2.62 and 1.50 MPa, respectively. This illustrated that the W/S was the key controlling factor for FS considering the effects of cement type, SP type and SP content. In conclusion, the addition of SP can increase the 28-day FS with different degrees, the reason might be that the addition of SP is helpful for improving the bending toughness of hardened SMCG. The 3-day and 7-day FSs were relatively satisfied, the 28-day FS was relatively low at the W/S of 1.5, regardless of cement type, SP type and SP content.

The reports about synergistic effects of MC type, SP type, SP content and W/S on the unconfined compressive strength (UCS) of SMCGs are rare at present. The UCS values of key formulations in Figure 13b are obtained to fill the corresponding gap in repair practices. Figure 13b shows the UCS of SMCG with key formulation at W/Ss of 1.0 and 1.5. For different formulations, there existed differences among UCS under different curing days. In comparison, the 28-day UCSs of CEM I SMCG were about 15.45 and 5.38 MPa when the W/Ss were 1.0 and 1.5, respectively. Although the addition of 1.2% PCE was helpful for improving the 28-day FS, the 1.2% PCE tended to decrease the 28-day UCS of SMCG. For example, with 1.2% PCE, the 28-day UCSs of CEM I, CEM II/B-M and CEM III/B were about 4.1–12.0, 4.2–13.0 and 4.0–12.9 MPa at the W/S of 1.0–1.5, respectively. Additionally, they were generally lower than that of CEM I SMCG without SP. Meanwhile, the 28-day UCS of CEM I decreased when the PCE content increased from 0.6% to 1.5%. This might be that the compactness tended to decrease with the increase in PCE content though the bleeding is little. With the addition of 1.0–2.0% N, it was observed that the UCS difference among 3-day, 7-day and 28-day was little, and the 1.5% N was more suitable to enhance the UCS. For example, the 3-day, 7-day and 28-day UCSs of CEM I SMCG at W/S of 1.0 were 9.9–11.0, 11.0–13.3 and 12.4–14.6 MPa when the content of N was 1.0–2.0%, respectively. Additionally, the 28-day UCSs were 13.9, 14.6 and 12.4 MPa when the N contents were 1.0%, 1.5% and 2.0%, respectively. Although the 28-day UCS (11.0 MPa) of CEM I SMCG with 1.2% M was lower than that with 1.5% N, 0.6% PCE or 0% SP, it was acceptable. The W/S was still the key controlling factor for UCS, it was found that the 28-day UCSs of various SMCG at the W/S of 1.5 were generally lower or close to the 3-day UCSs at the W/S of 1.0. For example, at the W/S of 1.5, the 28-day UCSs of SMCG with 0.6–1.5% PCE, 1.0–2.0% N and 1.2% M were about 4.1–5.2, 6.9–8.0 and 5.8 MPa, respectively. In conclusion, the SP reduced the 28-day UCS in different degrees.

**Figure 14:**
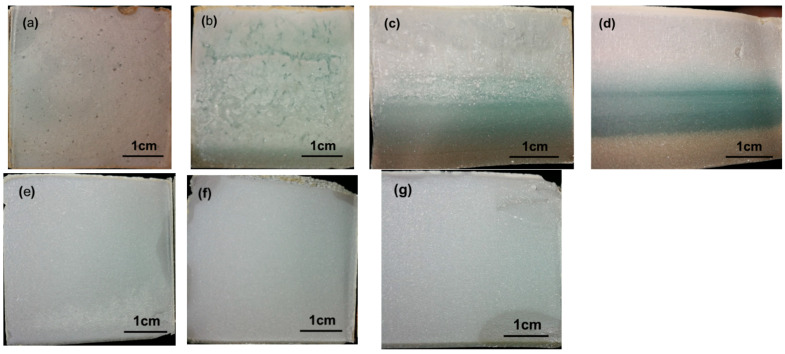
Failure section characteristics of the 28-day of CEM III/B MCG stone with various SP and W/S of 1.0 (**a**) 0.5%N (**b**) 1.0%N (**c**) 1.5%N (**d**) 2.0%N (**e**) 0.5%PCE (**f**) 1.0%PCE (**g**) 1.5%PCE.

Figure 14 shows the failure section characteristics of the 28-day of CEM III/B SMCG stone with various SP and W/S of 1.0. In Figure 14a–d, the color and hardening state varied significantly when the N content increased from 0.5% to 2.0%. For example, the hardening state started to become inhomogeneous when the N content exceeded 1.0%, there existed separated layers with corresponding colors when the N content reached 1.0%, and three separated layers existed obviously when the N content was 2.0%. This phenomenon might be mainly due to the different bleeding rate, hydration or hardening speed, and compatibility between SP and MC composition. In Figure 14 e–g, at the W/S of 1.0, there was almost no change of color and hardening state when the PCE content increased from 0.5% to 1.5%. Meanwhile, the height sizes were almost same as width sizes, proving there was almost no bleeding water, regardless of the PCE content. Within 0.5–1.5% content of PCE, it could be inferred that hardening or hydration rates are homogeneous, and the compatibility between PCE and MC composition is excellent.

**Figure 15:**
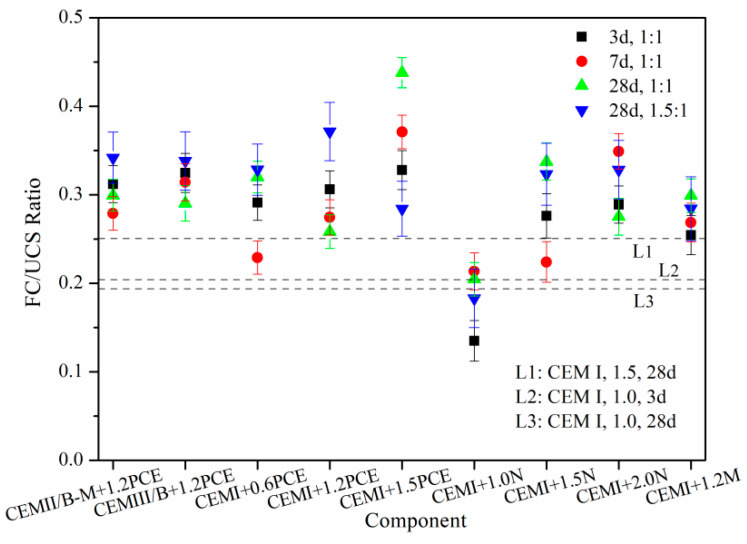
FS/USC ratio of SMCG with key formulation (cement type, SP type, SP content and W/S).

The studies about the brittleness evaluation of SMCG based on FS/UCS ratios are few at present, especially considering the combined effects of MC type, SP type, SP content and W/S. Figure 15 shows the FS/USC ratio of SMCG with key formulations. In comparison, the 3-day and 28-day FS/UCS ratios of CEM I SMCG were 0.20 and 0.19 at the W/S of 1.0, and the 28-day FS/UCS ratio was 0.25 at the W/S of 1.5. The 0.6–1.5% PCE, 1.5–2.0% N and 1.2% M can enhance the FS/UCS with different degrees; this might indicate that they have more bending or shearing resistance energy, and it is helpful for improving the brittleness of SMCG. The 28-day FS/UCS ratios of CEM I SMCG with 1.0% N were relatively low (0.13–0.21) at the W/S of 1.0 or 1.5; this might be because the 1.0% content of N is relatively low to improve the FS/UCS. It was observed that the FS/UCS tended to be affected seriously by UCS values because the UCS was generally much higher than FS. The effect of a single factor such as curing day, W/S, cement type or SP was not obvious and there existed some randomness. The FS/UCS or brittleness evaluation should be combined with the specific values of FS and UCS.

### 4.7. Microstructures

**Figure 16:**
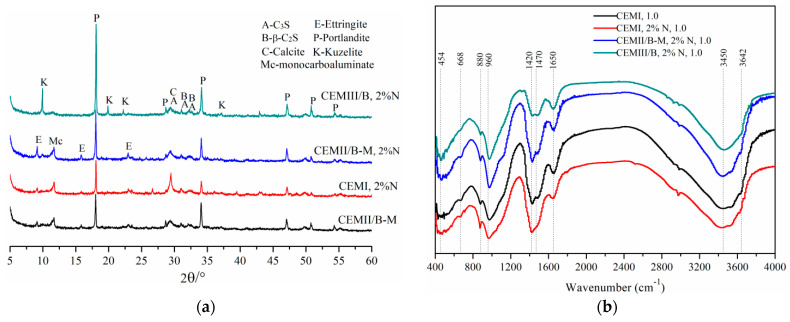
XRD and FTIR results of 28-day SMCG stone bodies at the W/S of 1.0. (**a**) XRD results; (**b**) FTIR results.

Figure 16 shows the XRD and FTIR results of 28-day SMCG stone bodies at the W/S of 1.0. In Figure 16a, the main hydration products of CEM II/B-M SMCG were Portlandite-syn Ca(OH)_2_, Calcium Silicate-Ca_3_SiO_5_, Larnite-beta-Ca_2_(SiO)_4_, Ettringite, etc. With 2% N, the main hydration products of CEM III/B SMCG were Portlandite-syn Ca(OH)_2_, Ettringite, AFm, Calcium Silicate-Ca_3_SiO_5_, Larnite-beta-Ca_2_(SiO)_4_, etc. The diffraction peak of ettringite was not found obviously, this might be because the ettringite had been transformed into AFm or reacted to amorphous gels, especially at initial W/S of 1.0. With 2% N, the main hydration products of CEM III/B SMCG were similar to those of CEM I SMCG. It was observed that there existed little difference of mineral composition between CEM II/B-M with 2% N and CEM II/B-M without SP. This might be because the 2% content of N was not easily found, the SP did not participate in mineral reaction or the organic polymer was difficult to detect through this method. Figure 16b shows the FTIR result of typical 28-day SMCG stone bodies at the W/S of 1.0. The vibration bands of the four typical samples were similar. The band appearing at about 454 cm^−1^ was attributed to the bending vibration of Si–O–Si and O–Si–O [46]. The band at about 668 cm^–1^ was assigned to the Al–O stretching vibration in AlO_4_ groups [47]. The bands at approximately 1420, 1470 and 880 cm^–1^ were associated to the asymmetric stretching mode of the O–C–O bonds of carbonates [47]. The band between 900 and 1300 cm^−1^ was related assigned to the asymmetric stretching mode of the Si–O–T (T: tetrahedral Si or Al) comprising the C–(A)–S–H gels [48]. The bands at about 1650 and 3450 cm^−1^ were features of water vibration, the intensity between 3450 and 3642 cm^−1^ tended to decrease with the increase in slag content, and the bands at around 3642 cm^−1^ could be attributed to the vibration of OH in Ca(OH)_2_ [49]. There existed other tiny shifted intensity peaks (454, 668, 880, 1470 and 3642 cm^−1^) among the selected SMCG formulation, this might be mainly due to the combined effects of slag and SP content.

**Figure 17:**
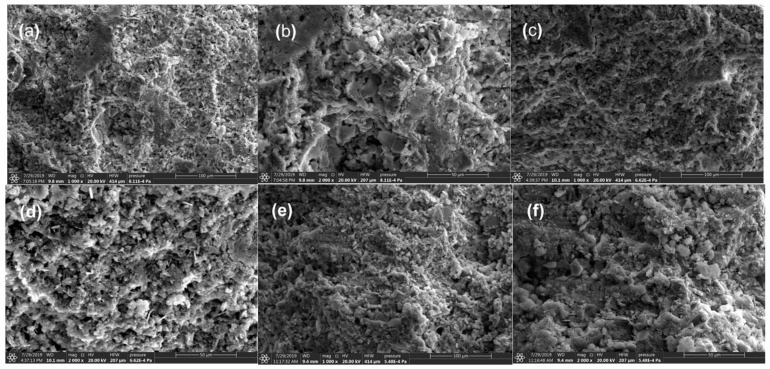
SEM photos of 28-day hardened SMCG at the W/S of 1.0 (**a**) CEM II/B-M, 1000× (**b**) CEM II/B-M, 2000× (**c**) CEM II/B-M, 1.5% N, 1000× (**d**) CEM II/B-M, 1.5% N, 2000× (**e**) CEM III/B, 1.5% N, 1000× (**f**) CEM III/B, 1.5% N, 2000×.

Figure 17 shows the SEM photos of 28-day hardened SMCG at the W/S of 1.0. In Figure 17a,b, the distribution of pore was random, the spaces among hydrated gels were relatively large. This kind of microstructure might not be helpful for the resistance to fracture. In Figure 17c,d, although there existed some microcracks, the addition of 2.0% N seemed to be helpful for improving the compactness of hydration minerals, and there were no significant big pores. In Figure 17e,f, the gel structures of CEM III/B SMCG with high slag content was compact with the dispersion effect 2.0% SP, and the gaps among minerals were small. In comparison with the results of FS and UCS, the 1.5% N could be helpful for improving the mechanical and microstructure performance of SMCG stone bodies. This might be because that the amount of 1.5% N was relatively approximate for improving the compactness and mechanical strength. Therefore, the optimal amount of SP can enhance the rheological behavior and improve the homogeneity of microstructure and mechanical performance.

## 5. Conclusions

(1) At the W/S of 1.0, the spreading ability and flowability of SMCG were relatively satisfied when the N, M and PCE contents were 1.5–2.0%, 1.2–1.8% and 0.9–1.5%, respectively.

(2) The effects of SP type and content on viscosity were more obvious than those of cement composition, the function mechanism of different SP was concluded, and the dispersion effects of SP were followed by PCE, M and N in order of influence degree.

(3) To avoid instability, long-setting (>24 h) or oversaturation, the W/S is suggested to be less than 1.5, and the N, M and PCE contents are suggested to be within 2.0%, 1.5% and 1.5% if the W/S exceeds 1.5.

(4) The W/S was the key controlling factor for FS and UCS, the 7-day and 3-day strengths were relatively satisfied at the W/S of 1.0, and the 3-day FS and UCS with the W/S of 1.0 tends to be higher than the 28-day ones with the W/S of 1.5.

(5) The SP could be helpful for improving the FS or brittleness, however, it tended to decrease UCS with various degrees. There is optimal SP content such as 1.5% N for UCS.

(6) The characteristics of microstructure and minerals were determined with SPs, and the optimal amount of SP could be helpful for improving the homogeneity of microstructure.

The optimized formulation of SMCG is important before engineering application. This research aimed at compensating for the missing data in existed reference and providing optimization suggestions for repair engineering.

## Figures and Tables

**Table 1:** The composition of different microfine cements in this study.

Cement Type		CEM I	CEM II/B-M	CEM III/B
Oxide (%)	SiO_2_	19.97	21.65	30.40
	Al_2_O_3_	4.02	9.15	9.80
	Fe_2_O_3_	3.38	2.85	1.50
	CaO	64.01	54.06	45.2
	MgO	2.36	3.90	5.90
	Na_2_O	0.21	0.24	0.46
	K_2_O	0.58	0.66	0.30
	SO_3_	2.09	3.62	2.80
	TiO_2_	0.28	0.49	0.50
	P_2_O_5_	0.21	0.23	0.18
	LOI	2.69	2.87	2.80
Components (%)	Clinker	90	63	25
	Limestone	5	5	3
	Slag	0	22	70
	Fly ash	0	5	0
	Gypsum	5	5	2

**Table 2:** The physical properties of different microfine cements in this study.

Cement Type	CEM I	CEM II/B-M	CEM III/B	OPC
*d*_10_ (µm)	0.89	1.01	1.06	2.39
*d*_50_ (µm)	3.21	3.64	4.22	9.70
*d*_95_ (µm)	10.92	11.15	12.81	37.62
*d*_100_ (µm)	20.02	20.02	20.50	110.00
Specific gravity	3.1	3.1	2.9	3.1
Fineness, m^2^/kg	870	850	825	430

**Table 3:** Property and classification of different SP used in this study.

Symbol	Type	Aspect	Bulk Density (kg/m^3^)	Solid Content (%)	pH	Recommended Dosage (%)	Repulsion Mechanism	
							Electrostatic	Steric Hindrance
N	FDN-C	Dark brown	1.2 × 10^3^	≥92	7–9	0.5–2.0	*	-
M	F10	White	0.5–0.8 × 10^3^	≥96	9–11.4	0.2–2.0	*	-
PCE	530P	White	0.5 ± 0.1 × 10^3^	≥95	7.0 ± 0.5	0.3–1.5	*	*

* represents the SP has the function of electrostatic repulsion mechanism.

**Table 4:** Mini-slumps and flow times of fresh SMCG under different W/S in this study.

W/S	Mini-Slump (mm), without SP				Flow Time (s), without SP			
	CEMI	CEMII/B-M	CEMIII/B	OPC	CEMI	CEMII/B-M	CEMIII/B	OPC
1:1	90	106	115	328	-	48.32	46.84	30.19
1.2:1	130	152	164	367	50.16	42.28	39.32	28.28
1.5:1	216	221	228	-	47.43	36.87	32.66	-
2:1	295	296	299	-	29.19	28.97	28.22	-
2.5:1	342	348	352	-	27.47	26.89	26.34	-

**Table 5:** Bleeding capacity of fresh SMCG with different SP, cement type and W/S in this study.

W/S	Bleeding Capacity (%), without SP				Bleeding Capacity (%), with SP				
	CEMI	CEMII/B-M	CEMIII/B	OPC	CEMI, 1.2%PCE	CEMI, 2.0%N	CEMI, 1.5%M	CEMII/B-M, 1.2%PCE	CEMIII/B, 1.2%PCE
1:1	0	0	0	29	0	0	0	1	2
1.2:1	1	1	1	32	1	1	1	2	4
1.5:1	3	3	5	38	5	8	17	6	10
2:1	14	16	20	-	16	24	23	21	24

## Data Availability

All data, models, and code generated or used during the study appear in the submitted article.

## References

[1] Sha F., Li S.C., Liu R.T., Li Z.F., Zhang Q.S. (2018). Experimental study on performance of cement-based grouts admixed with fly ash, bentonite, superplasticizer and water glass. Constr. Build. Mater..

[2] Sha F., Liu R.T., Li S.C., Lin C.J., Li Z.F., Liu B., Bai J.W. (2016). Application on different types of cementitious grouts for water-leakage operational tunnels. J. Cent. South. Univ..

[3] Sha F., Li S.C., Liu R.T., Zhang Q.S., Li Z.F. (2019). Performance of typical cement suspension-sodium silicate double slurry grout. Constr. Build. Mater..

[4] Sha F., Li S.C., Lin C.J., Liu R.T., Zhang Q.S., Yang L., Li Z.F. (2019). Research on penetration grouting diffusion experiment and reinforcement mechanism for sandy soil porous media. Rock. Soil. Mech..

[5] Sha F., Lin C.J., Li Z.F., Liu R.T. (2019). Reinforcement simulation of water-rich and broken rock with Portland cement-based grout. Constr. Build. Mater..

[6] Li S.C., Sha F., Liu R.T., Li W., Li Z.F., Wang G.C. (2017). Properties of cement-based grouts with high amounts of ground granulated blast-furnace slag and fly ash. J. Mater. Civil. Eng..

[7] Sha F., Li H.Y., Pan D., Liu H.L., Zhang X.F. (2020). Development of steel slag composite grouts for underground engineering. J. Mater. Res. Technol..

[8] Sha F., Fan G.X. (2021). Durability of a novel effective microfine cementitious grouting material in corrosion environments. Constr. Build. Mater..

[9] Sha F., Liu P. (2021). Development of high-performance microfine cementitious grout with high amount of fly ash, silica fume and slag. J. Mater. Civil. Eng..

[10] Saleh K., Mirza J., Ballivy G. (1993). Selection Criteria for Portland and Microfine Cement-Based Injection Grouts. Proceedings of the International Conference on Grouting in Rock and Concrete.

[11] Li S.C., Sha F., Liu R.T., Zhang Q.S., Li Z.F. (2017). Investigation on fundamental properties of microfine cement and cement-slag grouts. Constr. Build. Mater..

[12] Murat M., Yuksel Y. (2011). Engineering Properties of Medium-to-fine Sands Injected with Microfine Cement Grout. Mar. Georesour. Geotec..

[13] AFTES (Association Française des Travaux En Souterrain) (1998). Recommendations Concerning Grouting for Underground Structures Rehabilitation. Tunnels et Ouvrages Souterrains.

[14] Perret S., Palardy D., Ballivy G. (2000). Rheological behavior and setting time of microfine cement-based grouts. ACI Mater. J..

[15] Sha F., Jin Q., Liu P. (2020). Development of effective microfine cement-based grouts (EMCG) for porous and fissured strata. Constr. Build. Mater..

[16] BSI (British Standards Institution) (2000). Bleeding Rates, Sedimentation-(R6): The Bleeding (Sedimentation) Rate Shall be Determined in a Cylinder of 1000 ml Volume with an Inner Diameter of 60 mm. EN 12715.

[17] Yuan R.Z. (1996). Cementitious Materials.

[18] Pantazopoulos I.A., Markou I.N., Christodoulou D.N. (2012). Development of microfine cement grouts by pulverizing ordinary cements. Cem. Concr. Compos..

[19] Li S.C., Sha F., Liu R.T., Li Z.F., Zhang Q.S. (2017). Investigation on Viscous Behavior and Strength of Microfine Cement-based Grout Mixed with Microfine Fly Ash (MFA) and Superplasticizer (SP). Adv. Cem. Res..

[20] Sha F., Li S.C., Liu R.T., Li Z.F., Zhang Q.S. (2018). Effects of fineness on viscoelasticity of microfine cement-based grouts with fly ash, silica fume and superplasticizer. Adv. Cem. Res..

[21] Cunningham J.C., Dury B.L., Gregory T. (1989). Absorption Characteristics of Sulfonated Melamine Formaldehyde Condensates by High Performance Size Exclusion Chromatography. Cem. Concr. Res..

[22] Plank J., Hirsch C. (2007). Impact of Zeta Potential of Early Cement Hydration Phases on Superplasticizer Adsorption. Cem. Concr. Res..

[23] Yamada K., Hanehara S., Honma K. (2000). Effects of the Chemical Structure on the Properties of Polycarboxylate-type Superplasticizer. Cem. Concr. Res..

[24] Li C.Z., Feng N.Q., Li Y.D., Chen R.J. (2004). Effect of Polyethylene Oxide Chains on the Performance of Polycarboxylate-type Water-reducers. Cem. Concr. Res..

[25] Felekoğlu B., Sarıkahya H. (2008). Effect of Chemical Structure of Polycarboxylate-based Superplasticizers on Workability Retention of Self-compacting Concrete. Constr. Build. Mater..

[26] Uchikawa H., Hanebar S., Sawaki S. (1997). The Role of Steric Repulsive Force in the Dispersion of Ccement Particles in Fresh Paste Prepared with Organic Admixtures. Cem. Concr. Res..

[27] Langevin M.A. (1993). Rheological and Mechanical Behaviour of Microfine Cement-based Grouts. Master of Engineering.

[28] (2015). Standard Test Method for Slump of Hydraulic-Cement Concrete.

[29] (2016). Standard Test Method for Flow of Grout for Preplaced-Aggregate Concrete (Flow Vone Method).

[30] Izumo N., Koiwai A. Static Viscosity [SV] and Vibration Type Viscometer. Proceedings of the 23rd Sensing Forum.

[31] Izumo N., Koiwai A., Kazunaga U. (2009). Technological Background and Latest Market Requirements Concerning ‘Static Viscosity’ Measurement with A Tuning-Fork Vibration Viscometer. Proceedings of the Asia-Pacific Symposium on Measurement of Mass, Force and Torque.

[32] Banfill P.F.G., Carter R.E., Weaver P.J. (1991). Simultaneous Rheological and Kinetic Measurements on Cement Pastes. Cem. Concr. Res..

[33] Celik F., Canakci H. (2015). An Investigation of Rheological Properties of Cement-based Grout Mixed with Rice Husk Ash (RHA). Constr. Build. Mater..

[34] Izumo N., Oda H. (2008). Observation of Hardening Process by Static Viscosity Measurement of Cement Materials—Characterization of Phase Change from Paste to Solid Using the Tuning fork Vibration Type Viscosity Meter. Ceram. Jpn. Bull. Ceram. Soc. Jpn..

[35] Jefferis S.A., Lam C. (2012). Hydraulic characteristics of bentonite cake fabricated on cutoff walls. Clay. Clay. Miner..

[36] ASTM (2021). Standard Test Method for Linear Shrinkage and Coefficient of Thermal Expansion of Chemical-Resistant Mortars, Grouts, Monolithic Surfacings, and Polymer Concretes.

[37] (1999). Method of Testing Cements-Determination of Strength.

[38] Li Y.W., Yang C.L., Zhang Y.F., Zheng J., Guo H.L., Lu M.G. (2014). Study on Dispersion, Adsorption and Flow Retaining Behaviors of Cement Mortars with TPEG-type Polyether kind Polycarboxylate Superplasticizers. Constr. Build. Mater..

[39] Pourchet S., Liautaud S., Rinaldi D., Pochard I. (2012). Effect of the Repartition of the PEG Side Chains on the Adsorption and Dispersion Behaviors of PCEP in Presence of Sulfate. Cem. Concr. Res..

[40] Flatt R.J., Houst Y.F. (2001). A Simplified View on Chemical Effects Perturbing the Action of Superplasticizers. Cem. Concr. Res..

[41] Yoshioka K., Tazawa E., Kawai K., Enohata T. (2002). Adsorption Characteristics of Superplasticizers on Cement Component Minerals. Cem. Concr. Res..

[42] Zingg A., Winnefeld F., Holzer L., Pakusch J., Becker S., Figi R., Gauckler L. (2009). Interaction of Polycarboxylate-based Superplasticizers with Cements Containing Different C_3_A Amounts. Cem. Concr. Compos..

[43] Alrshoudi F., Mohammadhosseini H., Md Tahir M., Alyousef R., Alghamdi H., Alharbi Y., Alsaif A. (2020). Drying shrinkage and creep properties of prepacked aggregate concrete reinforced with waste polypropylene fibers. J. Build. Eng..

[44] Castaldo P., Palazzo B., Mariniello A. (2017). Effects of the axial force eccentricity on the time-variant structural reliability of aging r.c. cross-sections subjected to chloride-induced corrosion. Eng. Struct..

[45] Mehta P.K. (1986). Concrete: Structure, Properties and Materials.

[46] Nath S.K., Kumar S. (2019). Influence of Granulated Silico-Manganese Slag on Compressive Strength and Microstructure of Ambient Cured Alkali-Activated Fly Ash Binder. Waste. Biomass. Valori..

[47] Aydın S., Baradan B. (2013). Engineering Properties of Reactive Powder Concrete without Portland Cement. ACI Mater. J..

[48] Puertas F., Fernández-Jiménez A. (2003). Mineralogical and Microstructural Characterisation of Alkali-activated Fly Ash/slag Pastes. Cem. Concr. Compos.

[49] Puertas F., Palacios M., Vázquez T. (2006). Carbonation Process of Alkali-activated Slag Mortars. J. Mater. Sci..

